# Effect of time of day of recreational and household physical activity on prostate and breast cancer risk (MCC‐Spain study)

**DOI:** 10.1002/ijc.33310

**Published:** 2020-10-07

**Authors:** Jakob Weitzer, Gemma Castaño‐Vinyals, Nuria Aragonés, Inés Gómez‐Acebo, Marcela Guevara, Pilar Amiano, Vicente Martín, Ana Molina‐Barceló, Juan Alguacil, Victor Moreno, Claudia Suarez‐Calleja, José Juan Jiménez‐Moleón, Rafael Marcos‐Gragera, Kyriaki Papantoniou, Beatriz Pérez‐Gómez, Javier Llorca, Nieves Ascunce, Leire Gil, Esther Gracia‐Lavedan, Delphine Casabonne, Virginia Lope, Marina Pollán, Manolis Kogevinas

**Affiliations:** ^1^ Universitat Pompeu Fabra (UPF) Barcelona Spain; ^2^ ISGlobal Barcelona Spain; ^3^ CIBER Epidemiologia y Salud Pública (CIBERESP) Madrid Spain; ^4^ IMIM (Hospital del Mar Medical Research Institute) Barcelona Spain; ^5^ Environmental and Cancer Epidemiology Unit, National Center of Epidemiology Instituto de Salud Carlos III Madrid Spain; ^6^ Research Group on Statistics, Econometrics and Health (GRECS) University of Girona Girona Spain; ^7^ Unitat d'Epidemiologia i Registre de Càncer de Girona (UERCG), Pla Director d'Oncologia, Institut Català d'Oncologia, Institut d'Investigació Biomèdica de Girona (IdIBGi), Universitat de Girona Girona Spain; ^8^ Public Health Division, Department of Health Epidemiology Section Madrid Spain; ^9^ Cardiovascular & Metabolic Diseases Unit, National Centre for Epidemiology Carlos III Institute of Health Madrid Spain; ^10^ GEICAM Spanish Breast Cancer Group Madrid Spain; ^11^ Cancer and Public Health Area FISABIO—Public Health Valencia Spain; ^12^ Navarra Public Health Institute Pamplona Spain; ^13^ Navarra Institute for Health Research (IdiSNA) Pamplona Spain; ^14^ Universidad de Cantabria—IDIVAL Santander Spain; ^15^ IUOPA University of Oviedo and ISPA Oviedo Spain; ^16^ Ministry of Health of the Basque Government, Public Health Division of Gipuzkoa Biodonostia Health Research Institute Donostia‐San Sebastian Spain; ^17^ Department of Epidemiology, Center for Public Health Medical University of Vienna Vienna Austria; ^18^ Unit of Molecular and Genetic Epidemiology in Infections and Cancer (UNIC‐Molecular) Hospitalet de Llobregat Spain; ^19^ Cancer Epidemiology Research Program, IDIBELL Hospitalet de Llobregat Spain; ^20^ Catalan Institute of Oncology Hospitalet de Llobregat Spain; ^21^ Department of Clinical Sciences, Faculty of Medicine University of Barcelona Barcelona Spain; ^22^ The Research Group in Gene—Environment and Health Interactions (GIIGAS)/Institut of Biomedicine (IBIOMED), Universidad de León León Spain; ^23^ Faculty of Health Sciences, Department of Biomedical Sciences Area of Preventive Medicine and Public Health, Universidad de León León Spain; ^24^ Environmental Epidemiology and Neuroscience Laboratory RENSMA, Huelva University Huelva Spain; ^25^ Department of Preventive Medicine and Public Health, School of Medicine University of Granada & Instituto de Investigación Biosanitaria de Granada ibs.GRANADA Granada Spain

**Keywords:** breast, cancer, circadian disruption, physical activity, prostate

## Abstract

Experimental evidence indicates that exercise performed at different times of the day may affect circadian rhythms and circadian disruption has been linked to breast and prostate cancer. We examined in a population‐based case‐control study (MCC‐Spain) if the time‐of‐day when physical activity is done affects prostate and breast cancer risk. Lifetime recreational and household physical activity was assessed by in‐person interviews. Information on time‐of‐day of activity (assessed approximately 3 years after the assessment of lifetime physical activity and confounders) was available for 781 breast cancer cases, 865 population female controls, 504 prostate cases and 645 population male controls from 10 Spanish regions, 2008‐2013. We estimated odds ratios (ORs) and 95% confidence intervals (95% CI) for different activity timings compared to inactive subjects using unconditional logistic regression adjusting for confounders. Early morning (8‐10 am) activity was associated with a protective effect compared to no physical activity for both breast (OR = 0.74, 95% CI = 0.48‐1.15) and prostate cancer (OR = 0.73, 95% CI = 0.44‐1.20); meta‐OR for the two cancers combined 0.74 (95%CI = 0.53‐1.02). There was no effect observed for breast or prostate cancer for late morning to afternoon activity while a protective effect was also observed for evening activity only for prostate cancer (OR = 0.75, 95% CI = 0.45‐1.24). Protective effects of early morning activity were more pronounced for intermediate/evening chronotypes for both cancers. This is the first population‐based investigation identifying a differential effect of timing of physical activity on cancer risk with more pronounced effects for morning hour activity. Our results, if confirmed, may improve current physical activity recommendations for cancer prevention.

## What's new?

Exercise protects against a variety of cancers, but does time of day matter? Disrupting the body's circadian rhythm can boost cancer risk. Here, the authors compared breast and prostate cancer risk among people who exercised in the early morning, late morning, afternoon, and evening. They conducted a population‐based case‐control study, in which participants filled out a questionnaire about their patterns of sleeping, eating, and exercising. Exercising in the early morning appeared to be more strongly protective against breast and prostate cancer than exercising later in the day. Evening exercise appeared to have a moderate protective effect on prostate cancer.

**Abbreviations**
BMI: body mass index
CI: confidence interval
ER: estrogen receptor
HER2: human epidermal growth factor receptor 2
MCC‐Spain: Multicase‐Control Study‐Spain
MCTQ: Munich Chronotype Questionnaire
METs: metabolic equivalents of task
OR: odds ratio
PR: progesterone receptor

## 1 INTRODUCTION

Physical activity is an established protective factor for overall cancer risk^1^, ^2^, ^3^ and for specific major cancers such as colorectal and breast cancer. A recent meta‐analysis on breast cancer reported an approximate 20% reduction in risk associated with physical exercise for both premenopausal and postmenopausal women.^4^ Evidence for recreational physical activity and prostate cancer is less consistent although long‐term occupational physical activity seems to reduce prostate cancer risk.^5^

Circadian disruption results when the endogenous circadian rhythms are not in synchrony with environmental and social cues such as light exposure, work hours, diet and activity patterns and so forth. Exposure to artificial light at night, night shift work and mistimed diet may interfere with the normal nocturnal melatonin production and disrupt the circadian clock with numerous other biological consequences.^6^, ^7^, ^8^ Both breast and prostate cancer have been associated with different aspects of circadian disruption. Night shift work has been linked to an elevated cancer risk particularly in relation to breast and prostate cancer.^7^, ^8^ In 2007 the International Agency for Research on Cancer (IARC) classified shift work which includes circadian disruption as probably carcinogenic to humans (Group 2A)^9^ and a 2019 re‐evaluation reached the same conclusion.^10^ A diurnal pattern of diet has been associated with lower prostate and breast cancer risk,^11^, ^12^ while exposure to artificial light at night and particularly exposure to blue light spectrum light has been associated with higher breast and prostate cancer risk.^13^, ^14^

Chronotype is a human attribute that correlates with diurnal preference for activities in the morning or evening.^15^ Diurnal preference and chronotype may affect adaptation of circadian rhythms to new light‐dark conditions dictated by the use of artificial light, such as light at night exposure in night shift workers or light exposure and activity in the late evening/night due to a more nocturnal lifestyle in the general population. In a recent general population study examining circadian timings and chronotype, morning types had the highest protection when following diurnal patterns of diet compared to those having late supper (last evening meal).^12^

Mistimed physical activity could also disrupt circadian rhythms and therefore affect cancer risk and other health outcomes. Women doing less daily physical activity before noon (lowest quartile) had a higher odds ratio (OR) for obesity (1.26, 95% CI 1.05‐1.51) compared to women doing more physical activity in the morning.^16^ To our knowledge no study has investigated the timing of physical activity during the day (24 hours period), circadian disruption and cancer risk.

We examined the effect of timing of recreational physical activity on breast and prostate cancer risk in a population‐based case‐control study and possible effect modification by chronotype and shift work. We hypothesized that the beneficial effect of physical activity would be less pronounced for evening physical activity compared to morning activity.

## 2 MATERIAL AND METHODS

### 2.1 Study design, setting and population

MCC‐Spain is a population‐based case‐control study that includes five cancer types and 10 106 subjects (51.8% men).^17^ Data were collected between September 2008 and December 2013 in 23 hospitals (cases) and rosters of primary health care centers (controls) located in 12 Spanish provinces. For our study, we only considered breast (1738 cases) and prostate cancer (1112 cases) and 3403 population controls frequency matched by sex, age and region of residence.

Cases were between 20 and 85 years old, diagnosed according to the International Classification of Disease 10th Revision^18^ with female breast cancer (C50, D05.1, D05.7) or prostate cancer (C61, D07.5). Only histologically confirmed incident cases were included. Cases and controls were residing in the catchment area of the hospital for at least 6 months before the selection. Exclusion criteria were communication problems (mentally disabled, speech problems) and being physically disabled to participate in the study.

Incident cases were contacted at the hospitals while controls were randomly selected from records of primary health care centers. They were contacted by phone on behalf of their general practitioners. For each case, there was at least one control of the same sex and similar age (5‐year interval) randomly chosen out of five possible controls who were invited to a face‐to‐face interview. Response rates were 71% for breast (1750/2465) and 72% for prostate cancer (1115/1549) while 53% of the controls participated (4101/7743). Rates were calculated using interviewed subjects in the numerator and all subjects, including refusals, in the denominator.

**FIGURE 1:**
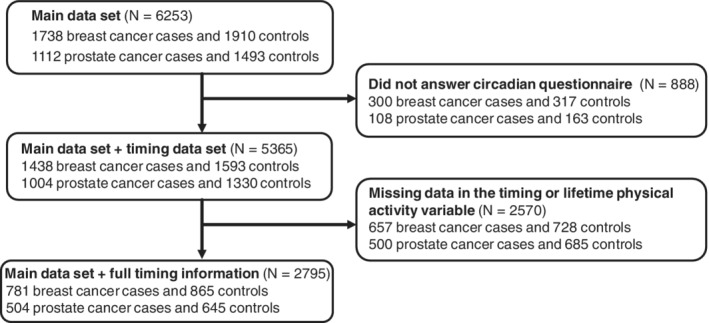
Flow chart describing exclusions and final sample size

For the current analyses, a sample of 5365 participants (breast cases: 1438, female controls: 1593; prostate cases: 1004, male controls: 1330) was used who had responded to the circadian timing questionnaire (Figure 1). Response rates were similar for cases and controls (breast cases: 82.7%, female control: 83.4%; prostate cases: 89.6%, male controls: 89.1%).

### 2.2 Data sources and variables

A computerized questionnaire (the main questionnaire), was administered by trained personnel in a face‐to‐face interview that took, on average, 70 minutes (range: 30‐130). Information was collected on residential history, personal and family medical history, sociodemographic factors, environmental exposures, occupational history, lifestyle (including all physical activity information used to estimate lifetime physical activity levels) and quality of the interview. Following the interview, anthropometric data and biological samples were taken. Immediately after the in‐person interview, a semiquantitative Food Frequency Questionnaire (response rate: 88%) was given to the participants, which was self‐administered by most of the participants; for a small percentage, an interviewer administered it when participants were unable to do it themselves. The questionnaire was based on a previously validated instrument in Spain.^19^ Between 6 months to 5 years (mean = 35 months [SD = 11]) later, breast and prostate cancer cases and controls were contacted by telephone to answer a circadian questionnaire including sleeping patterns, timing of food intake and physical activity (type, time‐of‐day, and age at start and end of activity), and more detailed information on shift work. Individual chronotype was assessed through the same follow‐up phone interview using the Munich Chronotype Questionnaire (MCTQ).

Clinical information was collected from medical records including tumor hormonal receptor status, differentiation grade and histological type. Breast cancer cases were classified in three subtypes according to hormonal receptors^20^, ^21^ (a) Progesterone receptor (PR) positive and/or estrogen receptor (ER) positive with luminal human epidermal growth factor receptor 2 (HER2) negative; (b) HER+ irrespective of PR and ER type; (c) triple negative with PR−, ER− and HER−. Prostate cancer cases were classified according to their biopsy Gleason score (a: Score ≤ 6; b: Score > 6). We did not use the latest grading system^22^ for the Gleason score because of a low sample size in some subgroups.

In the main and circadian questionnaire, participants reported every recreational physical activity done continuously for at least 6 months, throughout lifetime (from 5 years of age onward). The following question was used: “*We are going to ask you about any physical activity done outside working hours, including walking, any exercise, going to the gym, etc. We are interested in any physical activity you did continuously and for at least six months throughout your life. What activity do you do, or did you use to do*?” For each reported activity, a value of metabolic equivalents (METs) was assigned according to the Ainsworth's Compendium of Physical Activities.^23^ Furthermore, the duration of the activity through lifetime was estimated using the information on age, start and end of activity. In the main, but not in the circadian questionnaire, the frequency (h/week) of each activity was also assessed. For the analysis based on information provided in the circadian questionnaire, the timing pattern of the longest recreational activity done in lifetime for each person was chosen. For the sensitivity analysis the pattern of the timing for the most strenuous recreational activity done in lifetime was estimated. The most strenuous activity was defined as the activity with the highest METs assigned according to the Ainsworth's Compendium independent of the duration of the activity. Based on information provided in the main questionnaire we considered people as physically active in their lifetimes if they had done more than 1 MET * h/week as an annual average from 5 years of age onward with a lag of 1 year to the year of the interview. Only this variable, lifetime physical activity, was based on responses in the main questionnaire. All other physical activity variables in the present analysis are based on answers to the circadian questionnaire. After asking for the type of activity the following question was used to assess the time‐of‐day of activity: “*At what time do you do, or did you use to do this activity*?”. The available response‐categories were early morning (8‐10 am), late morning (10‐12 am), midday (12‐3 pm), afternoon (3‐7 pm), evening (7‐11 pm), night (11 pm‐8 am), “*no pattern*” and “*does not know*.” Categories were nonexclusive. Using these categories and taking into account frequency in each category a physical activity exposure variable was created using inactive people as the reference group: inactive, early morning (8‐10 am), late morning (10 am‐12 pm), midday to afternoon (12‐7 pm), evening (7‐11 pm) and other pattern (every other time or combination of times).

We considered as potential confounders age, education (a, less than primary school; b, primary school; c, secondary school; d, university), region of residence, tobacco consumption, obesity (body mass index [BMI] > 30), lifetime physical activity (average METs/week from 5 years of age onwards with a lag of 1 year to the year of the interview), and duration, intensity, and age at start and end of the longest (or most strenuous) activity. For women, additional possible confounders were menopausal status, defined as absence of menstruation during the last year, family history of breast cancer, age at menopause and menarche, estrogen intake, parity, age at first birth, and for men, ethnicity. Night work^10^ (a, Never night work: always day work + rotating no nights; b, night work: permanent night work + rotating night work; c, no work outside home) and chronotype (morning type, intermediate type, evening type^11^) were considered possible effect modifiers.

### 2.3 Final study population

Of the 5365 initial participants, 2795 respondents (52.1%) had full information on their activity pattern, lifetime physical activity, and key confounders age, education and region of residence (781 breast cases (54.3%), 865 female controls (54.3%) and 504 prostate cases (50.2%), 645 male controls (48.5%)) (Figure 1).

Respondents were slightly younger (respondent: median = 62, IQR: 52‐69; nonrespondent: median = 64, IQR: 54‐72), more likely to be female (respondent: 58.9% women; nonrespondent: 54.0%) and had a better education (respondent: 85.2% at least primary education; nonrespondent: 79.7%). In addition to missing data in the activity pattern, there were 31 subjects with missing values for chronotype, 35 for night work, eight for smoking status, 51 for family history of breast cancer, 50 for family history of prostate cancer, 74 for breast cancer subtype and six for Gleason score. Participants with missing values in these variables were not excluded to avoid reducing the sample size. However, they were excluded in the sensitivity analyses.

### 2.4 Statistical methods

A full‐case analysis was conducted including subjects without missing values (N = 2795 [52.1%]) for the main exposure and key confounders (age, region of residence and education). Inclusion of confounders in the models was based on a priori DAGs and change of effect estimates (≥10%). The covariates ethnicity, tobacco consumption, age at menopause and menarche, estrogen intake, and age at first birth did not change effect estimates. We estimated ORs with 95% confidence intervals using unconditional logistic regression. We adjusted for the frequency‐matched variables age (continuous) and region of residence (Madrid; Barcelona; Navarra; Guipuzcoa; Leon; Asturias; Huelva; Cantabria; Valencia; Granada; Gerona) for each cancer type separately, education (less than primary school; primary school; secondary school; university), and in women for menopausal status (premenopausal or postmenopausal) and family history of breast cancer (no family history of breast cancer; first degree relative; second degree relative; other degree relative). We also adjusted for lifetime average METs * h/week (continuous), METs (low: ≤4; intermediate: >4 and ≤6; high: >6), duration (continuous), and age at start and end of the longest done activity in lifetime (continuous), BMI (continuous), energy intake (kcal/day), and in women for parity (nulliparous: yes, no) in separate models. Combined cancer risk for breast and prostate cancers were calculated using STATA's metan function.

Analysis in subphenotypes was done for breast cancer hormone receptor types (hormone receptor positive, HER+ and triple negative), in premenopausal and postmenopausal women, and for prostate cancer according to the Gleason score (≤6 and >6). In addition, we ran stratified models for chronotype and night work to examine effect modification. We tested for multiplicative interactions with cross‐product terms and Wald test, and for additive interactions estimating RERI with STATA's lincom command.

Sensitivity analyses were conducted in participants without missing values in the variables (METs, age at start and end, duration of longest activity done in lifetime, lifetime annual average METs * h/week, parity, BMI and total energy intake) comparing the models with basic adjustment (age, region, education and for women menopausal status and family history of breast cancer) to models with additional adjustment (METs, age at start and end, duration of longest activity done in lifetime, lifetime annual average METs * h/week, parity, BMI and total energy intake). Furthermore, sensitivity analyses were conducted for all models a second time for the activity pattern of the most strenuous activity done in lifetime. In addition, we ran the main models again examining a category that included to the early morning group those who used to be active in the early morning and also at any other time of day. Due to missing values (170 in women and 24 in men) in the variables chronotype, night work, family history of breast cancer, breast cancer subtype and Gleason score we ran the main models again, restricted to participants without missing values in these variables. In further sensitivity analyses we set the activity threshold to >0.5 and >2.0 METs * h/week as an annual average to investigate how this would affect our results. We finally examined whether other circadian related variables (sleep and time of dinner‐last main meal) confounded the association of timing of physical activity with cancer. For all analysis the level of significance was set at two‐sided *P* < .05 and STATA (version 14.1, 2015, StataCorp LP) was used.

## 3 RESULTS

### 3.1 Study population characteristics

Breast cancer cases were younger than controls, more active in the evening (7‐11 pm) and had a higher percentage of night work and first‐degree family history of breast cancer. Prostate cancer cases were less educated, had a higher percentage of night work and first‐degree family history of prostate cancer and were more active in the late morning (10 am‐12 pm) and afternoon (3‐7 pm) compared to controls (Table 1).

**TABLE 1:** Descriptive characteristics of breast and prostate cancer cases and controls with a valid register of their activity pattern for the longest done physical activity in lifetime

	Breast cancer cases (N = 781)	Breast cancer controls (N = 865)	Prostate cancer cases (N = 504)	Prostate cancer controls (N = 645)
	N (%)	N (%)	N (%)	N (%)
Age (years); mean(SD)	55.3 (11.2)	58.2 (12.5)	65.9 (6.9)	65.9 (8.8)
Obesity	123 (15.7)	146 (16.9)	113 (22.4)	141 (21.9)
Education				
Less than primary school	88 (11.3)	157 (14.0)	122 (24.2)	94 (14.6)
Primary school	247 (31.6)	344 (30.4)	201 (39.9)	208 (32.2)
Secondary school	274 (35.1)	371 (32.8)	112 (22.2)	200 (31.0)
University	172 (22.0)	258 (22.8)	69 (13.7)	143 (22.2)
Smoking^a^				
Never	424 (54.6)	495 (57.3)	146 (29.1)	187 (29.1)
Current	175 (22.5)	181 (21.0)	109 (21.7)	127 (19.7)
Exsmoker	178 (22.9)	188 (21.7)	247 (49.2)	330 (51.2)
First degree family history of breast/prostate cancer^b^	112 (14.3)	82 (9.5)	94 (18.7)	42 (6.5)
Chronotype^c^				
Morning	295 (38.2)	312 (36.3)	249 (49.5)	320 (50.9)
Intermediate	301 (39.0)	356 (41.4)	175 (34.8)	232 (36.9)
Evening	176 (22.8)	192 (22.3)	79 (15.7)	77 (12.2)
Night work^d^	111 (14.5)	100 (11.9)	164 (32.6)	179 (27.8)
Menopause				
Premenopausal	275 (35.2)	262 (30.3)	NA	NA
Postmenopausal	506 (64.8)	603 (69.7)	NA	NA
Physical activity pattern				
Inactive	132 (16.9)	143 (16.5)	62 (12.3)	67 (10.4)
Early morning (8‐10 am)	55 (7.0)	78 (9.0)	64 (12.7)	90 (14.0)
Late morning (10 am‐12 pm)	140 (17.9)	168 (19.4)	103 (20.4)	98 (15.2)
Midday (12‐3 pm)	20 (2.6)	15 (1.7)	7 (1.4)	17 (2.6)
Afternoon (3‐7 pm)	101 (12.9)	110 (12.8)	35 (6.9)	31 (4.8)
Evening (7‐11 pm)	132 (16.9)	128 (14.8)	63 (12.5)	101 (15.7)
Night (11 pm‐8 am)	2 (0.3)	7 (0.8)	4 (0.8)	6 (0.9)
No pattern^e^	91 (11.7)	86 (10.0)	60 (12.0)	105 (16.2)
Other pattern^e^	108 (13.8)	130 (15.0)	106 (21.0)	130 (20.2)
Lifetime annual average of METs * h/week				
≤1 MET	132 (16.9)	143 (16.5)	62 (12.3)	67 (10.4)
to 7.9 METs	334 (42.8)	434 (50.2)	204 (40.5)	274 (42.5)
8 to 16 METs	163 (20.9)	145 (16.8)	104 (20.6)	133 (20.6)
>16 METs	152 (19.4)	143 (16.5)	134 (26.6)	171 (26.5)

Abbreviation: NA, not applicable.

^a^ Missing for four breast cancer cases (0.5%) and one control (0.1%); two prostate cancer cases (0.4%) and one controls (0.16%).

^b^ Missing for 18 breast cancer cases (2.3%) and 33 controls (3.8%); 16 prostate cancer cases (3.2%) and 43 controls (5.2%).

^c^ Missing for nine breast cancer cases (0.1%) and five controls (0.5%); one prostate cancer case (0.2%) and 16 controls (2.5%).

^d^ Missing for 13 breast cancer cases (1.7%) and 21 controls (2.44%); one prostate cancer case (0.2%).

^e^ In the no pattern category participants indicated having no pattern while the other pattern category includes all patterns that did not exactly match with the above mentioned categories.

One hundred and twenty‐nine men (11.2%) and 275 women (16.7%) reported a lifetime annual average of 1 MET * h/week. Overall, being more active through lifetime was moderately associated (after adjusting for age and education) with a reduction in prostate (OR = 0.85, CI 95% = 0.57‐1.26) while no or a minimal effect was observed for breast cancer risk (OR = 0.97, 95% CI = 0.74‐1.26).

Concerning the longest done physical activity in lifetime, the most frequently reported activities were walking (47.6%), going to the gym (10.3%), swimming (7.2%), soccer (4.3%), riding the bike (3.6%), other intermediate intensity activities equal to 4.5 METs (3.4%) and household chores, gardening, dancing, excursions, playing tennis and other low intensity activities equal to 3 METs (2%, respectively). Subjects active in the early morning were more likely to engage in swimming or riding the bike compared to the other groups. Walking and other low intensity activities were more frequent in subjects active in the late morning. Going to the gym, playing soccer, dancing, playing tennis and other intermediate intensity activities were more frequent in subjects active in the evening. The most frequently reported time‐of‐day of activity was the late morning (10 am‐12 pm) and evening (7‐11 pm) (Table 1).

Most subjects were adults (median age = 38, IQR: 20‐54) when they began their longest‐achieved activity in lifetime and stopped when becoming older adults (median age = 59, IQR: 46‐67). Breast cancer cases began their longest‐achieved activity at a similar time in life (median age = 35, IQR: 20‐46) compared to female controls (median age = 35, IQR: 20‐50) but stopped slightly earlier in life (median age = 53, IQR. 44‐62) than female controls (median age = 56, IQR: 45‐66). Prostate cancer cases and male controls began (case: median age = 43.5, IQR: 20‐60; control: median age = 43.5, IQR: 20‐60) and stopped their longest‐done activity at a similar age (case: median age = 64, IQR:53‐69; control: median age = 64, IQR: 50‐70)).

### 3.2 Effect of timing of physical activity of the longest done physical activity in lifetime

Early morning (8‐10 am) activity was associated with a protective effect compared to no physical activity for both breast (OR = 0.74, 0.48‐1.15) and prostate cancer (OR = 0.73, 0.44‐1.20) but confidence intervals were wide. The meta‐OR for the two cancers combined for early morning exercise was 0.74 (0.53‐1.02). There was no effect observed for breast or prostate cancer for late morning or midday to afternoon physical activity while a moderate protective effect was also observed for evening physical activity but only for prostate cancer (OR = 0.75, 0.45‐1.24). Male subjects with other patterns including both morning, evening and night activity, showed also a protective pattern similar to morning physical activity (OR = 0.79, 0.52‐1.21) (Table 2).

**TABLE 2:** Association of breast and prostate cancer with timing of the longest done physical activity in lifetime

	Breast cancer cases (N = 781)	Breast cancer controls (N = 865)		Prostate cancer cases (N = 504)	Prostate cancer controls (N = 645)	
	N (%)	N (%)	OR (95% CI)^a^ ^,^ ^b^	N (%)	N (%)	OR (95% CI)^a^
Inactive	132 (16.9)	143 (16.5)	1	62 (12.3)	67 (10.4)	1
Early morning (8‐10 am)	55 (7.0)	78 (9.0)	0.74 (0.48–1.15)	64 (12.7)	90 (14.0)	0.73 (0.44‐1.20)
Late morning (10 am‐12 pm)	140 (17.9)	168 (19.4)	0.96 (0.68‐1.36)	103 (20.4)	98 (15.2)	1.12 (0.69‐1.80)
Midday to afternoon (12‐7 pm)	121 (15.5)	125 (14.5)	1.03 (0.72‐1.47)	42 (8.3)	48 (7.4)	1.11 (0.45‐1.23)
Evening (7‐11 pm)	132 (16.9)	128 (14.8)	1.10 (0.77‐1.57)	63 (12.5)	101 (15.7)	0.75 (0.45–1.24)
Other pattern^c^	201 (25.7)	223 (25.8)	0.96 (0.70‐1.32)	170 (33.7)	241 (37.4)	0.79 (0.52‐1.21)

^a^ Adjusted for age, education and region.

^b^ Further adjusted for menopause and family history of breast cancer (51 missing, missing for 18 breast cancer cases (2.3%) and 33 controls (3.8%).

^c^ Includes all participants active during the night (11 pm‐8 am), who had no pattern or another pattern (see Table 1).

When conducting the analyses using participants who had complete data on additional confounders, adjustment for METs (two missings), age at start and end, and duration of longest activity done in lifetime (60 missings), lifetime annual average METs * h/week, parity (one missing), BMI and total energy intake (232 missings) only minor changes in the ORs were found as compared to the previous analyses that had adjusted for age, education, region and for women also menopause and family history (Supporting Information Table 1).

### 3.3 Modification by chronotype and night work

For breast cancer, the protective effect of early morning activity was more pronounced among intermediate and evening chronotypes with ORs of 0.55 (0.25‐1.20) and 0.53 (0.14‐1.99) respectively, although the number of subjects in some strata was very small (Table 3). The same pattern was observed for prostate cancer (intermediate: OR = 0.64, 0.27‐1.56; evening: OR = 0.45, 0.09‐2.15) but confidence intervals were wide. For breast cancer, ORs for physical activity among evening types were generally low irrespective of the time of the day of the activity (Table 3).

**TABLE 3:** Association of breast and prostate cancer with timing of the longest done physical activity in lifetime by chronotype (31 missings)

	Breast cancer cases (N = 781)	Breast cancer controls (N = 865)		Prostate cancer cases (N = 504)	Prostate cancer controls (N = 645)	
	N (%)	N (%)	OR (95% CI)^a^ ^,^ ^b^	N (%)	N (%)	OR (95% CI)^a^
Morning chronotype						
Inactive	50 (17.0)	50 (16.0)	1	27 (10.8)	35 (10.9)	1
Early morning (8‐10 am)	35 (11.9)	38 (12.2)	0.96 (0.51‐1.83)	40 (16.1)	55 (17.2)	0.92 (0.46‐1.83)
Late morning (10 am‐12 pm)	52 (17.6)	59 (18.9)	0.96 (0.54‐1.72)	41 (16.5)	50 (15.6)	0.97 (0.47‐2.00)
Midday to afternoon (12‐7 pm)	36 (12.2)	45 (14.4)	0.85 (0.46‐1.58)	17 (6.8)	25 (7.8)	1.01 (0.44‐2.33)
Evening (7‐11 pm)	39 (13.2)	41 (13.1)	0.89 (0.47‐1.69)	35 (14.1)	44 (13.8)	1.14 (0.56‐2.34)
Other pattern^c^	83 (28.1)	79 (25.3)	1.15 (0.68‐1.97)	89 (35.7)	111 (34.7)	1.05 (0.57‐1.93)
Intermediate chronotype						
Inactive	43 (14.3)	62 (17.4)	1	25 (14.3)	24 (10.3)	1
Early morning (8‐10 am)	14 (4.6)	32 (9.0)	0.55 (0.25–1.20)	18 (10.3)	24 (10.3)	0.64 (0.27–1.56)
Late morning (10 am‐12 pm)	62 (20.6)	73 (20.5)	1.36 (0.77‐2.39)	45 (25.7)	34 (14.7)	1.31 (0.60‐2.83)
Midday to afternoon (12‐7 pm)	52 (17.3)	47 (13.2)	1.55 (0.86‐2.80)	12 (6.9)	16 (6.9)	0.82 (0.30‐2.22)
Evening (7‐11 pm)	55 (18.3)	49 (13.8)	1.52 (0.84‐2.77)	17 (9.7)	39 (16.8)	0.48 (0.20‐1.17)
Other pattern^c^	75 (24.9)	93 (26.1)	1.02 (0.60‐1.73)	58 (33.1)	95 (41.0)	0.63 (0.32‐1.26)
Evening chronotype						
Inactive	37 (21.0)	30 (15.6)	1	9 (9.4)	7 (9.1)	1
Early morning (8‐10 am)	5 (2.8)	8 (4.2)	0.53 (0.14–1.99)	7 (7.3)	9 (11.7)	0.45 (0.09‐2.15)
Late morning (10 am‐12 pm)	26 (14.8)	35 (18.2)	0.09 (0.23‐1.10)	22 (22.9)	12 (15.6)	1.20 (0.31‐4.64)
Midday to afternoon (12‐7 pm)	33 (18.8)	33 (17.2)	0.65 (0.31‐1.39)	15 (15.6)	6 (7.8)	2.22 (0.48‐10.3)
Evening (7‐11 pm)	37 (21.0)	36 (18.8)	0.87 (0.42‐1.78)	14 (14.6)	17 (22.1)	0.46 (0.11‐1.87)
Other pattern^c^	38 (21.6)	50 (26.0)	0.55 (0.27‐1.11)	29 (30.2)	26 (33.7)	0.67 (0.19‐2.32)

^a^ Adjusted for age, education and region.

^b^ Further adjusted for menopause and family history of breast cancer (51 missing, missing for 18 breast cancer cases (2.3%) and 33 controls (3.8%)).

^c^ Includes all participants active during the night (11 pm‐8 am), who had no pattern or another pattern (see Table 1).

Additional adjustment for other potential confounding factors, did not alter the overall risk pattern although it led to a similar effect of early morning activity in women with an early or intermediate chronotype (Supporting Information Table 2). This difference was mainly due to the change (reduction) in the number of subjects because of the exclusion of subjects with missing values rather than to an effect of adjustment. Additional adjustment, however, did seem to reduce risk differences across time‐of‐day in men with an early chronotype.

Similar effects were found in people who never worked at night, with the most consistent effects observed for early morning activity for both breast (OR = 0.70, 95% CI = 0.42‐1.14) and prostate cancer (OR = 0.61, 95% CI = 0.34‐1.09) (Supporting Information Table 3). Results for other activity times were similar to those found for all subjects (Table 2, Supporting Information Table 3). Effect modification by chronotype and night work was not statistically significant, neither on a multiplicative nor on an additive scale. Furthermore, chronotype and night work did not confound the association between time‐of‐day of physical activity and cancer risk.

### 3.4 Subphenotype analysis

When analyzing clinical subgroups in breast cancer (Table 4) early morning (8‐10 am) activity seemed to be protective for the estrogen/progestogen receptor positive and the HER+ subgroup. Triple negative breast cancer risk was similar across categories of physical activity timing. The moderate protective effect of early morning activity tended to be stronger for postmenopausal women compared to premenopausal women. (Table 4).

**TABLE 4:** Association (OR, 95% CI) between breast cancer and timing of the longest done physical activity in lifetime by menopausal status and hormone receptor (N = cases; 10% of cases had no information on hormone receptor)

	Hormonal receptor positive (N = 528)^a^ ^,^ ^b^ ^,^ ^c^	HER+ (N = 126)^a^ ^,^ ^b^ ^,^ ^c^	Triple negative (N = 53)^a^ ^,^ ^b^ ^,^ ^c^	Premenopausal (N = 275)^a^ ^,^ ^c^	Postmenopausal (N = 506)^a^ ^,^ ^c^
Inactive	1	1	1	1	1
Early morning (8‐10 am)	0.76 (0.46‐1.26)	0.37 (0.15‐0.93)	1.33 (0.42‐4.13)	0.93 (0.43‐1.98)	0.62 (0.35‐1.09)
Late morning (10 am‐12 pm)	1.05 (0.71‐1.55)	0.60 (0.31‐1.16)	1.28 (0.49‐3.34)	0.90 (0.44‐1.85)	1.04 (0.69‐1.56)
Midday to afternoon (12‐7 pm)	1.06 (0.71‐1.60)	0.73 (0.37‐1.42)	1.19 (0.43‐3.28)	1.26 (0.67‐2.37)	0.94 (0.60‐1.47)
Evening (7‐11 pm)	1.31 (0.88‐1.97)	0.73 (0.38‐1.41)	1.17 (0.41‐3.34)	0.96 (0.54‐1.72)	1.19 (0.74‐1.93)
Other pattern^d^	1.09 (0.76‐1.57)	0.70 (0.39‐1.25)	0.84 (0.31‐2.23)	0.77 (0.43‐1.37)	1.04 (0.70‐1.55)

^a^ Adjusted for age, education and region.

^b^ Further adjusted for menopause.

^c^ Family history of breast cancer (51 missing, missing for 18 breast cancer cases (2.3%), and 33 controls (3.8%)).

^d^ Includes all participants active during the night (11 pm‐8 am), who had no pattern or another pattern (see Table 1).

In subtype analysis, the effects were similar between clinical subtypes of prostate cancer (Gleason score ≤ 6 vs Gleason score 7 or higher) with a moderate protective effect observed for early morning and evening activity (Table 5), similar to the overall effect (Table 2). However, the protective effect of early morning physical activity tended to be slightly stronger for aggressive tumors (Gleason score 7 or higher).

**TABLE 5:** Association (OR, 95% CI) between prostate cancer and timing of the longest done physical activity in lifetime by Gleason score (N = cases)

	Gleason score ≤ 6 (N = 237)^a^	Gleason score > 6 (N = 261)^a^
Inactive	1	1
Early morning (8‐10 am)	0.80 (0.43‐1.47)	0.69 (0.37‐1.29)
Late morning (10 am‐12 pm)	0.89 (0.48‐1.63)	1.32 (0.74‐2.36)
Midday to afternoon (12‐7 pm)	1.14 (0.56‐2.31)	1.06 (0.52‐2.15)
Evening (7‐11 pm)	0.71 (0.38‐1.32)	0.79 (0.42‐1.49)
Other pattern^b^	0.87 (0.52‐1.45)	0.69 (0.41‐1.17)

^a^ Adjusted for age, education and region.

^b^ Includes all participants active during the night (11 pm‐8 am), who had no pattern or another pattern (see Table 1).

### 3.5 Sensitivity analyses

The analysis of the most strenuous activity done in lifetime instead of the longest activity lead to similar results in women but not in men. (Supporting Information Table 4). When setting the activity threshold to >0.5 METs * h/week risk estimates were lower across all timing categories (Supporting Information Table 5), being more active (after adjusting for age and education) was associated with lower cancer risk (breast cancer: OR = 0.89, 95% CI = 0.64‐1.24; prostate cancer: OR = 0.88, 95% CI = 0.55‐1.40). When setting it to >2.0 METs * h/week estimates were higher (Supporting Information Table 6), being more active was not associated with a lower cancer risk (breast cancer: OR = 1.24; 95% CI = 0.99‐1.53; prostate cancer: OR = 1.05, 95% CI = 0.78‐1.42). Nonetheless, early morning activity was always linked to the strongest protective effect in women. The same applied for early morning and evening activity in men. Examining a category that included in the morning group those who used to be active in the early morning and also at any other time of day, revealed the same pattern as found in the main analysis, although less pronounced (Supporting Information Table 7). Restricting the models to participants who did not have missing values in the variables chronotype, night work, family history of breast cancer, breast cancer subtype and Gleason score did not change effect estimates (Supporting Information Table 8).

We finally examined whether other circadian related variables, specifically sleep and timing of dinner (last main meal) could confound the association of timing of physical activity with cancer risk. To evaluate confounding we limited the analysis to those without missing values, and adjusted for sleep duration (in hours, <7, 7‐8, >8) and for time of dinner (≤9:30 pm, >9:30 pm—the median dinner time in this population). The ORs not adjusting for sleep or timing of diet are comparable to those in Table 2 with minor differences because of the change in the numbers. There was no confounding by sleep or timing of dinner with minimal changes in the ORs (Supporting Information Table 9).

## 4 DISCUSSION

In the present study, we observed that the overall protective effect of recreational and household physical activity for cancer may vary depending on the time of the day of the activity. We found that early morning activity might be more protective than late morning‐afternoon activity for both breast and prostate cancer risk. Findings on evening activity differed with a moderate protective effect observed only for prostate cancer. There was no consistent pattern by chronotype, and differences were observed in tumor subphenotypes. The biological pathways associated with a differential effect of physical activity during the day are unclear and may be related to circadian hormonal patterns.

Mean lifetime physical activity levels were very low in the present study compared to other populations^24^, ^25^, ^26^ and this complicates comparison of our results to other research. Most other studies compared participants who did not do any physical activity to participants who did any physical activity or compared the least active to the most active quartile.^4^, ^5^, ^24^, ^25^, ^26^ In contrast, participants in our reference group reported some activity throughout their lifetime. To our knowledge there is no other study that investigated the time‐of‐day of activity in relation to cancer risk. However, some evidence already exists on activity at different ages in life and cancer risk.^27^

Timing of physical activity has been associated with changes in physiological parameters related to circadian rhythms. In one experiment, evening exercise delayed the falling phase of the circadian rhythm of plasma melatonin in men, reduced rapid eye movement sleep, slowed down the decline of rectal temperature and accelerated the heart rate during the sleep of the following night. Morning exercise increased the number of heart waves during the sleep. This could indicate that morning exercise stimulates parasympathetic activity, while evening exercise promotes sympathetic activity during the following night sleep.^28^ Another study reported significantly higher plasma interleukin(IL)‐6 and adrenaline levels in men after evening exercise (5‐6 pm) compared to morning exercise (9‐10 am).^29^ IL‐6, a pleiotropic cytokine, has anti‐inflammatory (cis signaling) and proinflammatory characteristics (trans signaling)^30^ and during and after exercise IL‐6 has positive effects.^31^, ^32^ Morning exercise was associated with a lower number of sleep stage‐shifts over the whole night and a lower number of wake stages during the second half of the night.^33^ A later peak of the body temperature rhythm (acrophase delay) and a lower amplitude (smaller difference between the peak and the mean value of the wave of the circadian rhythm) was reported in an evening exercise group (9 pm) compared to morning exercise (9 am).^34^ This evidence hints toward an effect of time‐of‐day of physical activity on circadian disruption which was linked to tumorigenesis.^35^

In our study, the moderate protective effect of breast cancer associated with early morning activity compared to evening activity may be related to a different effect of timing of physical activity on sex steroid production. Higher levels of estrogens are associated with increased breast cancer risk.^36^ Physical activity is associated with lower estrogen levels^37^ and the estradiol production peaks around 7 am.^38^ Morning activity compared to evening activity might reduce estradiol levels shortly after the morning peak of the cycle. This approach could also explain why early morning activity does not seem to be more protective than evening activity for the triple negative breast cancer subtype, since it is estrogen and progesterone hormone factor negative. There are no prior studies examining the time‐of‐day effect of physical activity on sex steroids but in a previous study we found increased progestogen levels in night workers compared to day workers.^39^

Effects of physical activity on melatonin levels could also provide clues for a differential effect of time‐of‐day of activity and the protective effect of early morning activity. Melatonin has a broad variety of anticarcinogenic effects^40^ and noon and/or afternoon exercise were shown to delay the on‐set and acrophase (peak) of the melatonin rhythm.^41^ Thus, late midday to afternoon (12‐7 pm) compared to early morning (8‐10 am) activity could delay the onset and peak of melatonin production and may lead to a shorter period of melatonin production and reduced melatonin levels. Melatonin is also known to reduce estrogen levels,^42^ and therefore light induced or physical activity induced suppression of melatonin may in turn lead to increased sex steroid levels. Therefore, melatonin may mediate some of the suggested effects of physical activity on sex hormone production. Furthermore, the peak level of the melatonin rhythm decreases with age.^43^ The delaying effect of afternoon and noon exercise on the melatonin rhythm could therefore have a stronger effect in older people; however, the cancer risk related to midday‐afternoon activity did not seem bigger in postmenopausal women and for prostate cancer with a Gleason score of 7 or higher.

The protective effect of early morning compared to midday to afternoon activity was stronger in intermediate and late chronotypes. The later onset of melatonin production in intermediate and late chronotypes^44^ could be perhaps affected to a greater extend by midday to afternoon exercise, leading to a larger reduction of melatonin production because its synthesis is limited by daylight in the morning.^45^ Last, the discrepancy between the effect of evening activity on breast and prostate cancer risk could also be explained by melatonin rhythm disruption. Yamanaka et al^28^ report a delay of the falling phase of the melatonin rhythm in men after exercise in the evening. This delay might ultimately lead to a higher overall production of melatonin and consequently reduce cancer risk.

Obesity has been associated with both breast and prostate cancer and may be an additional pathway through which physical activity and timing of physical activity may be associated with lower cancer risk. Higher weight loss^46^ and lower total calorie consumption^47^ have been observed among persons doing morning exercise compared to exercise in other hours of the day. However, in our analysis, adjusting for BMI and total calorie consumption did not change the cancer risk pattern by timing category.

Major strengths of the study are the population‐based design and the large sample size. Loss of statistical power due to missing values and exposure misclassification are the main limitations of the study. For all risk estimates confidence intervals were wide and numbers were small in stratified analyses. Only lifetime recreational and household physical activity was considered, mainly done during adulthood. Occupational physical activity was not assessed, and results might therefore be confounded. We did not analyze household and recreational activity separately although they might have a differential effect on cancer risk. Misclassification of exposure may have occurred due to inherent difficulties in evaluating physical activity in epidemiologic studies.^48^ This type of bias typically tends to attenuate findings and may have been even more pronounced in the evaluation of timing of activity. The threshold for inactivity in lifetime was set at a very low level (1 MET * h/week as annual average) and was selected to ensure a sufficient group size for the reference and exposure groups in analysis. Although the overall risk pattern (eg, morning activity more protective than afternoon activity) did not disappear when using a different inactivity threshold in sensitivity analyses, effect estimates did change. In addition, because we tested a novel hypothesis, there is limited knowledge on measurement of the main exposure variable (timing of activity patterns). Although the questionnaire used was detailed, the validity of the questions is not known, nor is its repeatability in different populations. We collected timing of physical activity information on average 35 months (SD = 11) after the administration of the main questionnaire. Given the good prognosis of both cancers, it is unlikely that this may have resulted to biased results due to selective cancer survival due to physical activity. However, recall bias and reverse causation could have biased our results and results should therefore be interpreted with caution. Finally, we did not control for two potential confounders, diet and sleep patterns.

Overall our findings indicate that time of the day of physical activity is an important aspect of physical activity that may potentiate the protective effect of physical activity on cancer risk. The effect of timing of physical activity on cancer risk should be examined in future research with a more detailed assessment of activity patterns, also including occupational activity. More evidence on biological mechanisms of how timing of physical activity influences circadian rhythms is needed and the proposed mechanisms regarding the potential effect of timing on cancer risk should be further examined.

## CONFLICT OF INTEREST

The authors state to have no conflict of interest.

## ETHICS STATEMENT

National and international guidelines (Declaration of Helsinki and Ethical code) were adhered to and data were managed according to the Spanish Law on Data Confidentiality (Ley Orgánica 15/1999 de 13 de Diciembre de Protección de Datos de carácter personal [LOPD]) eliminating personal identifiers. The objectives of the study were explained to each participant, followed by the signing of an informed consent. All ethics committees of the involved institutions approved the MCC‐Spain study protocol.

## Supporting information


**Data S1** Supporting information.

## Data Availability

Data are available by contacting the corresponding author and following acceptance by the contributing centers (https://www.mccspain.org/).
