# Supplementary material for: Effect of time of day of recreational and household physical activity on prostate and breast cancer risk (MCC‐Spain study)
Source: Int J Cancer. 2020 Oct 7;148(6):1360–71. doi: 10.1002/ijc.33310 (PMC7891656; doi:10.1002/ijc.33310)
Supplement: Supplementary file 1 — Data S1 Supporting information. [file IJC-148-1360-s001.pdf]

Supplementary material

Title. Effect of time of day of recreational and household physical activity on prostate and breast cancer risk (MCC-Spain Study)

Authors

Jakob Weitzer, Gemma Castaño-Vinyals, Nuria Aragonés, Inés Gómez-Acebo, Marcela Guevara, Pilar Amiano, Vicente Martín, Ana Molina-Barceló, Juan Alguacil, Victor Moreno, Claudia Suarez-Calleja, José Juan Jiménez-Moleón, Rafael Marcos-Gragera, Kyriaki Papantoniou, Beatriz Pérez-Gómez, Javier Llorca, Nieves Ascunce, Leire Gil, Esther Gracia-Lavedan, Delphine Casabonne, Virginia Lope, Marina Pollán, Manolis Kogevinas

Table of contents

Supplemental Table 1. Association of breast and prostate cancer with timing of the longest done physical activity in lifetime. Comparison of models for participants without missing values in other possible confounders.

Supplemental Table 2. Association of breast and prostate cancer with timing of the longest done physical activity in lifetime by chronotype. Comparison of models for participants without missing values in other possible confounders.

Supplemental Table 3. Association of breast and prostate cancer with timing of the longest done physical activity in lifetime in people who never worked at night (people who never had worked outside their home are not included).

Supplemental Table 4. Association of breast and prostate cancer with timing of the most strenuous physical activity done in lifetime

Supplement Table 5. Association of breast and prostate cancer with timing of the longest done physical activity in lifetime (Participants were considered Inactive if they had done ≤ 0.5 METs * hours per week as an annual average from 5 years of age onward with a lag of 1 year to the interview)

Supplement Table 6. Association of breast and prostate cancer with timing of the longest done physical activity in lifetime (Participants were considered Inactive if they had done ≤ 2 METs * hours per week as an annual average from 5 years of age onward with a lag of 1 year to the interview)

Supplement Table 7. Association of breast and prostate cancer with timing of the longest done physical activity in lifetime (the early morning exercise category also includes those who used to exercise in the early morning and also at any other time)

Supplement Table 8. Association of breast and prostate cancer with timing of the longest done physical activity in lifetime in participants without missing values in the variables chronotype, night work, family history of breast cancer, and breast cancer and prostate cancer subtype.

Supplement Table 9. Association of breast and prostate cancer with timing of the longest done physical activity in lifetime adjusting also for sleep or for timing of dinner (last main meal). Analysis limited to subjects without missing information.

Supplemental Table 1**.** Association of breast and prostate cancer with timing of the longest done physical activity in lifetime. Comparison of models for participants without missing values in other possible confounders.

	**Breast cancer OR** (95 % CI) *N*=1470[1] **[a,b]**	**Breast cancer OR** (95 % CI) *N*=1470[[1] **[a,b,c,d]**	**Breast cancer OR** (95 % CI) *N*=1470[[1] **[a,b,c,d,e]**	**Prostate cancer OR** (95 % CI) *N*=1037[2] **[a]**	**Prostate cancer OR** (95 % CI) *N*=1037 [2] **[a,d]**	**Prostate cancer OR** (95 % CI) *N*=1037 [2] **[a,d,e]**
Inactive	1	1	1	1	1	1
Early morning (8-10am)	0.78 (0.48-1.25)	0.76 (0.47-1.25)	0.77 (0.47-1.26)	0.72 (0.42-1.24)	0.62 (0.35-1.10)	0.62 (0.35-1.10)
Late morning (10am-12pm)	1.01 (0.70-1.46)	0.99 (0.67-1.46)	1.03 (0.69-1.52)	1.08 (0.65-1.80)	1.02 (0.60-1.74)	1.02 (0.60-1.74)
Midday to afternoon (12-7pm)	1.10 (0.75-1.61)	1.07 (0.72-1.60)	1.06 (0.71-1.58)	1.31 (0.71-2.40)	1.10 (0.58-2.09)	1.10 (0.58-2.09)
Evening (7-11pm)	1.32 (0.90-1.94)	1.28 (0.86-1.92)	1.30 (0.87-1.95)	0.69 (0.41-1.18)	0.54* (0.31-0.96)	0.53* (0.30-0.94)
Other pattern[3]	1.08 (0.77-1.52)	0.97 (0.66-1.41)	0.97 (0.66-1.42)	0.76 (0.49-1.20)	0.66 (0.40-1.08)	0.66 (0.40-1.07)

[a] adjusted for age, education, region [b] menopause, family history of breast cancer [c] parity [d] METs, duration, and age at start and end of longest done activity in lifetime and lifetime annual average METs*hrs/week [e] total energy intake and BMI [1] women without missing values in the set of variables for further adjustment: 1 missing values in parity, 50 women did not indicate the duration of their only reported activity, 129 missing values in total energy intake, 1 missing values in Mets of longest activity done in lifetime [2] men without missing values in the set of variables for further adjustment: 10 men did not indicate the duration of their only reported activity, 103 missing values in total energy intake, 1 missing values in Mets of longest activity done in lifetime [3] includes all participants active during the night (11pm-8am), who had no pattern or another pattern (see Table.1)

Supplemental Table 2. Association of breast and prostate cancer with timing of the longest done physical activity in lifetime by chronotype. Comparison of models for participants without missing values in other possible confounders.

	**Breast cancer OR** (95 % CI) [a,b]	**Breast cancer OR** (95 % CI) [a,b,c]	**Breast cancer OR** (95 % CI) [a,b,c,d,e]	**Prostate cancer OR** (95 % CI) [a]	**Prostate cancer OR** (95 % CI) [a,d]	**Prostate cancer OR** (95 % CI) [a,d,e]
**Morning chronotype**	*N*=549 [1]	*N*=549 [1]	*N*=549 [1]	*N*=512 [2]	*N*=512 [2]	*N*=512 [2]
Inactive	1	1	1	1	1	1
Early Morning (8-10am)	0.86 (0.43-1.72)	0.92 (0.44-1.92)	0.88 (0.42-1.85)	0.90 (0.42-1.87)	0.76 (0.35-1.64)	0.71 (0.33-1.56)
Late morning (10am-12pm)	1.05 (0.57-1.94)	1.16 (0.61-2.21)	1.20 (0.62-2.32)	0.89 (0.47-1.94)	0.84 (0.37-1.85)	0.79 (0.35-1.76)
Midday to afternoon (12-7pm)	0.86 (0.45-1.64)	0.93 (0.47-1.86)	0.88 (0.43-1.77)	1.16 (0.47-2.84)	0.93 (0.37-2.37)	0.89 (0.34-2.28)
Evening (7-11pm)	0.93 (0.48-1.81)	1.02 (0.50-2.07)	1.02 (0.50-2.09)	1.01 (0.47-2.13)	0.72 (0.32-1.64)	0.67 (0.29-1.53)
Other pattern[3]	1.21 (0.69-2.12)	1.33 (0.70-2.50)	1.27 (0.67-2.43)	1.03 (0.54-1.97)	0.88 (0.43-1.79)	0.67 (0.40-1.68)
**Intermediate chronotype**	*N*=581 [1]	*N*=581 [1]	*N*=581 [1]	*N*=366 [2]	*N*=366 [2]	*N*=366 [2]
Inactive	1	1	1	1	1	1
Early Morning (8-10am)	0.82 (0.35-1.90)	0.72 (0.30-1.73)	0.73 (0.30-1.75)	0.61 (0.24-1.59)	0.60 (0.21-1.70)	0.55 (0.19-1.58)
Late morning (10am-12pm)	1.49 (0.80-2.76)	1.21 (0.63-2.34)	1.21 (0.63-2.34)	1.19 (0.53-2.69)	1.14 (0.49-2.69)	1.13 (0.48-2.66)
Midday to afternoon (12-7pm)	1.90 (1.00-3.60)	1.61 (0.83-3.12)	1.62 (0.83-3.14)	0.88 (0.31-2.50)	0.92 (0.30-2.81)	0.93 (0.30-2.85)
Evening (7-11pm)	2.11 (1.11-4.01)	1.73 (0.88-3.39)	1.73 (0.88-3.41)	0.46 (0.18-1.16)	0.42 (0.16-1.12)	0.421 (0.15-1.11)
Other pattern[3]	1.24 (0.70-2.23)	0.91 (0.48-1.73)	0.92 (0.48-1.74)	0.53 (0.25-1.10)	0.49 (0.22-1.10)	0.50 (0.22-1.12)
**Evening chronotype**	*N*=330 [1]	*N*=330 [1]	*N*=330 [1]	*N*=136 [2]	*N*=136 [2]	*N*=136 [2]
Inactive	1	1	1	1	1	1
Early Morning (8-10am)	0.42 (0.10-1.87)	0.37 (0.07-1.91)	0.37 (0.07-1.91)	0.51 (0.07-3.50)	0.41 (0.05-3.59)	0.44 (0.05-3.9)
Late morning (10am-12pm)	0.47 (0.20-1.11)	0.51 (0.20-1.29)	0.55 (0.22-1.40)	2.13 (0.47-9.5)	1.95 (0.38-10.0)	1.96 (0.37-10.0)
Midday to afternoon (12-7pm)	0.66 (0.29-1.50)	0.57 (0.24-1.36)	0.55 (0.23-1.33)	3.9 (0.67-22.6)	3.6 (0.53-23.8)	3.88 (0.57-26.4)
Evening (7-11pm)	1.14 (0.52-2.53)	1.05 (0.45-2.48)	1.08 (0.45-2.59)	0.44 (0.09-2.07)	0.31 (0.05-1.82)	0.31 (0.05-1.83)
Other pattern[3]	0.67 (0.31-1.44)	0.46 (0.20-1.10)	0.49 (0.20-1.17)	0.87 (0.22-3.4)	0.71 (0.14-3.69)	0.74 (0.14-3.9)

[a] adjusted for age, education and region [b] menopause and family history of breast cancer [c] parity [d] METs, duration, and age at start and end of longest done activity in lifetime and lifetime annual average Mets*hrs/week [e] total energy intake and BMI [1] women without missing values in the set of variables for further adjustment: 1 missing values in parity, 50 women did not indicate the duration of their only reported activity, 129 missing values in total energy intake, 1 missing values in Mets of longest activity done in lifetime [2] men without missing values in the set of variables for further adjustment: 10 men did not indicate the duration of their only reported activity, 103 missing

values in total energy intake, 1 missing values in Mets of longest activity done in lifetime [3] in cludes all participants active during the night (11pm-8am), who had no pattern or another pattern (see Table.1)

Supplemental Table 3. *Association of breast and prostate cancer with timing of the longest done physical activity in lifetime in people who never worked at night (people who never had worked outside their home are not included).*

	**Breast cancer cases** (*N*=601)	**Breast cancer controls** (*N*=635)	**OR** (95 % CI) [a,b]	**Prostate cancer cases** (*N*=339)	**Prostate cancer controls** (*N*=466)	**OR** (95 % CI) [a]
**Detailed category**	N (%)	N (%)		N (%)	N (%)	
Inactive	107 (17.8)	105 (16.5)	1	49 (14.5)	56 (12.0)	1
Early Morning (8-10am)	42 (7.0)	63 (9.9)	0.70 (0.42-1.14)	40 (11.8)	68 (14.6)	0.61 (0.34-1.09)
Late morning (10am-12pm)	111 (18.5)	119 (18.7)	1.01 (0.68-1.50)	67 (19.8)	72 (15.5)	1.02 (0.59-1.77)
Midday to afternoon (12-7pm)	82 (13.6)	87 (13.7)	0.91 (0.59-1.39)	29 (8.6)	36 (7.7)	1.01 (0.52-1.95)
Evening (7-11pm)	108 (18.0)	96 (15.1)	1.07 (0.71-1.61)	47 (13.9)	75 (16.1)	0.76 (0.43-1.35)
Other pattern[3]	151 (25.1)	165 (26.0)	0.92 (0.64-1.32)	107 (31.6)	159 (34.1)	0.77 (0.47-1.23)

[a] adjusted for age, education and region [b] menopause and family history of breast cancer (33 missing, for 13 breast cancer cases (2.2%) and 20 controls (3.1%)) [3] includes all participants active during the night (11pm-8am), who had no pattern or another pattern (see Table.1)

Supplemental Table 4. Association of breast and prostate cancer with timing of the most strenuous physical activity done in lifetime [1]

	**Breast cancer cases** (*N*=786)	**Breast cancer controls** (*N*=900)	**OR** (95 % CI) [a,b]	**Prostate cancer cases** (*N*=495)	**Prostate cancer controls** (*N*=670)	**OR** (95 % CI) [a]
	N (%)	N (%)		N (%)	N (%)	
Inactive	136 (17.3)	178 (19.8)	1	64 (12.9)	101 (15.1)	1
Early morning (8-10am)	66 (8.4)	93 (10.3)	0.92 (0.61-1.38)	67 (13.5)	81 (12.1)	1.39 (0.87-2.23)
Late morning (10am-12pm)	144 (18.3)	161 (17.9)	1.26 (0.90-1.76)	87 (17.6)	96 (14.3)	1.46 (0.92-2.30)
Midday to afternoon (12-7pm)	116 (14.8)	133 (14.8)	1.11 (0.78-1.58)	39 (7.9)	51 (7.6)	1.50 (0.87-2.60)
Evening (7-11pm)	154 (19.6)	154 (17.1)	1.19 (0.85-1.67)	83 (16.8)	125 (18.7)	1.27 (0.81-1.99)
Other pattern	170 (21.6)	181 (20.1)	1.17 (0.85-1.62)	155 (31.3)	216 (32.2)	1.20 (0.81-1.77)

[a] adjusted for age, education and region [b] menopause (2 missing) and family history of breast cancer (71 missing, 25 breast cancer cases (2.5%) and 46 controls (4.0%)) [1] number of cases and controls are slightly different compared to the primary analysis (longest done activity in lifetime) because some participants had information on the timing pattern of the longest done activity but not on the most strenuous and vice versa.

Supplement Table 5. Association of breast and prostate cancer with timing of the longest done physical activity in lifetime (Participants were considered Inactive if they had done ≤ 0.5 METs * hours per week as an annual average from 5 years of age onward with a lag of 1 year to the interview)

	**Breast cancer cases** (*N*=781)	**Breast cancer controls** (*N*=865)	**OR** (95 % CI) [a,b]	**Prostate cancer cases** (*N*=504)	**Prostate cancer controls** (*N*=645)	**OR** (95 % CI) [a]
	N (%)	N (%)		N (%)	N (%)	
Inactive	82 (10.5)	78 (9.0)	1	40 (7.9)	39 (6.1)	1
Early morning (8-10am)	65 (8.3)	84 (9.7)	0.75 (0.47-1.20)	70 (13.9)	94 (14.6)	0.68 (0.38-1.20)
Late morning (10am-12pm)	155 (19.9)	188 (21.7)	0.88 (0.59-1.31)	108 (21.4)	106 (16.4)	0.94 (0.53-1.64)
Midday to afternoon (12-7pm)	129 (16.5)	132 (15.3)	0.97 (0.64-1.45)	43 (8.5)	48 (7.4)	0.97 (0.51-1.85)
Evening (7-11pm)	142 (18.2)	143 (16.5)	0.96 (0.64-1.44)	65 (12.9)	105 (16.3)	0.64 (0.36-1.14)
Other pattern[1]	208 (26.6)	240 (27.8)	0.84 (0.58-1.23)	178 (35.2)	253 (39.2)	0.68 (0.41-1.14)

[a] adjusted for age, education and region [b] further adjusted for menopause and family history of breast cancer (51 missing, missing for 18 breast cancer cases (2.3%) and 33 controls (3.8%) [1] includes all participants active during the night (11pm-8am), who had no pattern or another pattern (see Table.1)

Supplement Table 6. Association of breast and prostate cancer with timing of the longest done physical activity in lifetime (Participants were considered Inactive if they had done ≤ 2 METs * hours per week as an annual average from 5 years of age onward with a lag of 1 year to the interview)

	**Breast cancer cases** (*N*=781)	**Breast cancer controls** (*N*=865)	**OR** (95 % CI) [a,b]	**Prostate cancer cases** (*N*=504)	**Prostate cancer controls** (*N*=645)	**OR** (95 % CI) [a]	**Pooled OR** (95 % CI)
	N (%)	N (%)		N (%)	N (%)		
Inactive	206 (26.4)	258 (29.8)	1	98 (19.4)	122 (18.9)	1	
Early morning (8-10am)	48 (6.2)	64 (7.4)	0.94 (0.61-1.46)	61 (12.1)	83 (12.9)	0.84 (0.54-1.32)	
Late morning (10am-12pm)	123 (16.8)	138 (16.0)	1.20 (0.87-1.66)	90 (17.9)	90 (14.0)	1.15 (0.74-1.78)	
Midday to afternoon (12-7pm)	105 (13.4)	102 (11.8)	1.27 (0.90-1.79)	38 (7.5)	40 (6.2)	1.27 (0.73-2.19)	
Evening (7-11pm)	113 (14.5)	112 (13.0)	1.23 (0.88-1.73)	59 (11.7)	91 (14.1)	0.85 (0.53-1.34)	
Other pattern[1]	186 (23.8)	191 (22.1)	1.22 (0.92-1.63)	158 (31.4)	219 (34.0)	0.89 (0.63-1.27)	

[a] adjusted for age, education and region [b] further adjusted for menopause and family history of breast cancer (51 missing, missing for 18 breast cancer cases (2.3%) and 33 controls (3.8%) [1] includes all participants active during the night (11pm-8am), who had no pattern or another pattern (see Table.1)

Supplement Table 7. Association of breast and prostate cancer with timing of the longest done physical activity in lifetime (the early morning exercise category also includes those who used to exercise in the early morning and also at any other time)

	**Breast cancer cases** (*N*=781)	**Breast cancer controls** (*N*=865)	**OR** (95 % CI) **[a,b]**	**Prostate cancer cases** (*N*=504)	**Prostate cancer controls** (*N*=645)	**OR** (95 % CI) **[a]**
	N (%)	N (%)		N (%)	N (%)	
Inactive	132 (16.9)	143 (16.5)	1	62 (12.3)	67 (10.4)	1
Early morning (8-10am)	116 (14.9)	150 (17.3)	0.85 (0.60-1.21)	122 (24.2)	154 (23.9)	0.85 (0.54-1.32)
Late morning (10am-12pm)	140 (17.9)	168 (19.4)	0.96 (0.68-1.35)	103 (20.5)	98 (15.2)	1.13 (0.54-1.32)
Midday to afternoon (12-7pm)	121 (15.5)	125 (14.5)	1.03 (0.72-1.47)	42 (8.3)	48 (7.4)	1.11 (0.63-1.97)
Evening (7-11pm)	132 (16.9)	128 (14.8)	1.10 (0.77-1.58)	63 (12.5)	101 (15.7)	0.75 (0.45-1.24)
Other pattern[1]	140 (17.9)	151 (17.5)	0.96 (0.68-1.36)	112 (22.2)	177 (27.4)	0.72 (0.46-1.11)

[a] adjusted for age, education and region [b] further adjusted for menopause and family history of breast cancer (51 missing, missing for 18 breast cancer cases (2.3%) and 33 controls (3.8%) [1] includes all participants active during the night (11pm-8am), who had no pattern or another pattern (see Table.1)

Supplement Table 8. Association of breast and prostate cancer with timing of the longest done physical activity in lifetime in participants without missing values in the variables chronotype, night work, family history of breast cancer, and breast cancer and prostate cancer subtype.

	**Breast cancer cases** (*N*=670)	**Breast cancer controls** (*N*=806)	**OR** (95 % CI) **[a,b]**	**Prostate cancer cases** (*N*=496)	**Prostate cancer controls** (*N*=629)	**OR** (95 % CI) **[a]**
	N (%)	N (%)		N (%)	N (%)	
Inactive	109 (16.39	130 (16.1)	1	62 (12.5)	66 (10.5)	1
Early morning (8-10am)	46 (6.9)	75 (9.3)	0.70 (0.43-1.11)	64 (12.9)	88(14.0)	0.73 (0.44-1.20)
Late morning (10am-12pm)	121 (18.1)	159 (19.7)	0.96 (0.66-1.39)	101 (20.4)	96 (15.3)	1.12 (0.69-1.81)
Midday to afternoon (12-7pm)	101 (15.1)	113 (14.0)	1.00 (0.68-1.48)	42 (8.5)	47 (7.5)	1.11 (0.63-1.98)
Evening (7-11pm)	121 (18.1)	121 (15.0)	1.21 (0.83-1.77)	62 (12.5)	100 (15.9)	0.74 (0.44-1.22)
Other pattern[1]	172 (25.7)	208 (25.8)	1.00 (0.71-1.41)	165 (33.3)	232 (36.9)	0.79 (0.52-1.21)

[a] adjusted for age, education and region [b] further adjusted for menopause and family history of breast cancer (51 missing, missing for 18 breast cancer cases (2.3%) and 33 controls (3.8%) [1] includes all participants active during the night (11pm-8am), who had no pattern or another pattern (see Table.1)

Supplement Table 9. Association of breast and prostate cancer with timing of the longest done physical activity in lifetime adjusting also for sleep or for timing of dinner (last main meal). Analysis limited to subjects without missing information.

	Breast, Sleep N=1567[a, b]		Prostate, Sleep N=1072[a]	
	OR not adjusting sleep	OR adjusting sleep	OR not adjusting sleep	OR adjusting sleep
Early morning	0.77 (0.49-1.21)	0.77 (0.49-1.22)	0.69 (0.41-1.16)	0.71 (0.42-1.19)
Late morning	0.99 (0.70-1.42)	1.00 (0.70-1.42)	1.13 (0.69-1.86)	1.14 (0.69-1.87)
Midday to afternoon	1.03 (0.71-1.49)	1.03 (0.71-1.49)	1.19 (0.65-2.18)	1.21 (0.66-2.22)
Evening	1.16 (0.80-1.67)	1.16 (0.80-1.68)	0.70 (0.42-1.17)	0.70 (0.42-1.17)
Other pattern	1.02 (0.74-1.42)	1.02 (0.74-1.42)	0.79 (0.51-1.22)	0.79 (0.51-1.23)
	Breast, timing of diet N=1382 [a, b]		Prostate, timing of diet N=787 [a]	
	OR not adjusting timing of diet	OR adjusting timing of diet	OR not adjusting timing of diet	OR adjusting timing of diet
Early morning	0.75 (0.46-1.20)	0.75 (0.46-1.20)	0.55 (0.30-1.00)	0.55 (0.31-1.00)
Late morning	0.97 (0.67-1.41)	0.97 (0.67-1.41)	1.04 (0.59-1.81)	1.04 (0.60-1.83)
Midday to afternoon	0.96 (0.65-1.43)	0.96 (0.65-1.43)	0.96 (0.50-1.87)	0.96 (0.49-1.87)
Evening	1.16 (0.79-1.70)	1.16 (0.79-1.70)	0.73 (0.41-1.31)	0.73 (0.41-1.31)
Other pattern	0.99 (0.70-1.39)	0.99 (0.70-1.39)	0.75 (0.46-1.22)	0.75 (0.46-1.23)

[a] adjusted for age, education and region [b] further adjusted for menopause and family history of breast cancer
